# Blockchain and non-fungible tokens (NFTs) in surgery: Hype or hope?

**DOI:** 10.1016/j.sipas.2022.100065

**Published:** 2022-02-26

**Authors:** Sai Batchu, Owen S. Henry, Karan Patel, Abraham Hakim, Umur Atabek, Francis R. Spitz, Young K. Hong

**Affiliations:** a Cooper Medical School at Rowan University, Camden, NJ, USA; b Department of Surgery, Cooper University Hospital, Camden NJ, USA

Blockchain technology and non-fungible tokens (NFTs) are making headlines beyond the realms of art and finance, demonstrating opportunities for adoption in other contexts [1]. Briefly, blockchain is a generic term used to describe a type of shared append-only database that stores information digitally, where chunks of data are stored periodically as blocks and cryptographically chained together. Blockchains are unique due to their immutability, transparency, and decentralization. When discussing NFTs, non-fungible is an economic term describing assets that are not interchangeable. For example, while $1 USD can be exchanged for another fungible $1 USD, artworks cannot be exchanged due to their unique properties and are therefore non-fungible. Token describes the ability of an NFT to represent digital or physical objects on the blockchain. Therefore, an NFT is simply a blockchain representation showing the existence of any tangible or intangible asset (i.e., a certificate of authenticity and ownership for a unique digital record of an asset).

Although the concepts of digitally representing assets through unique identifications are not novel, using tamper-resistant blockchain technology allows NFTs to become potent solutions for problems involving efficiency, transparency, and permanence. While the unique construction of NFTs have allowed them to become ideal vehicles to digitally represent assets in the fields of art and finance [2], [3], [4], their application to healthcare is not beyond imagination. With NFTs, anything may be registered to the blockchain and permanently assigned a digital representation. The tamper-resistant technology allows NFTs to solve problems involving efficiency and privacy, both cardinal concerns in any health system. Blockchain solutions to digital processes such as blood banking and the sharing of patient health information seem like logical first steps [5], [6], [7], [8]. However, more procedural based medical disciplines such as surgery may be overlooked as potential beneficiaries of this new technology. Nonetheless, the application of NFTs in surgery deserves exploration.

Perhaps the most apparent benefit of NFTs in surgery is more efficient storage of patient surgical data. Although using blockchain alone may be useful in organizing such records, the unique qualities of NFTs can optimize patient-level data access. The conversion of patient data such as surgical history, laboratory values, imaging records, and surgeon's notes into a single digital asset removes the surgeon's dependence on multiple parties to access a patient's clinical profile. Whenever surgeons manage acutely ill patients, they require proper and timely access to medical histories. The field of transplant surgery combines both these priorities with rigorous patient-identification standards and allograft selection. A blockchain and NFT-based system may help organize reliable access of patient data for transplant surgeons and patients undergoing organ transplant. Moreover, a decentralized transplant database would allow all parties to monitor the transplantation process while also mitigating risk of fraud and increasing efficiency by eliminating unnecessary intermediators [9, 10].

Representing a patient's pertinent surgical data with NFTs can also allow for more rapid communication between surgeons and patients. Various surgical groups often use different electronic health record systems which tend to lack interoperability. As a result, patient data is not only fragmented across the different systems but transferring data between surgeons regarding the same patient is hindered. In an NFT-based system, the owners of a particular health record NFT would receive a digital file and retain full ownership. Accordingly, the owner could be the patient, with surgeons adding metadata (attributes pertaining to the information in the NFT) directly to the NFT, or vice-versa. The metadata amendments could include changes in current medication regimens, additional imaging studies, and information about recent or upcoming surgeries. The NFT metadata can then be seamlessly transferred to other surgeons involved in the patient's care without need for complex bureaucratic affairs and troublesome navigation of noncompatible health record systems.

Alongside the rising utilization of telehealth in surgery, NFTs and blockchain may be employed to improve data security and patient privacy. By design, telehealth is an electronic arena where sensitive patient health information is shared. It is therefore inherently vulnerable to ethical, legal, and social issues [11, 12]. Telehealth carries many benefits for surgeons, particularly exemplified during the COVID-19 pandemic [13]. These benefits may be strengthened with the use of NFTs, as they are specifically designed with exclusive digital certificate-based access to enhance security and privacy. Patient encounters, surgical videos, and remote surgeon education all present opportunities for NFTs to enhance the quality of surgeon communication.

With concerns regarding data security and patient privacy come possible impacts on the economics of healthcare. In a proposed blockchain and NFT-based system, health data becomes decentralized and belongs more in the hands of each individual patient rather than solely with the healthcare institutions [10, 14]. Blockchain and NFT applications may however allow direct combination of clinical outcomes measurements with cost and reimbursement calculations, shifting toward a more value-based economic model. Overall, feasibility may demand balancing this type of disruption with integration into the current system.

Robotic surgical systems are also becoming more prevalent, allowing surgeons to operate on patients remotely via wireless connections. However, the possibility of malicious actors to compromise telesurgeries by affecting network connections has raised concerns about security [15]. Blockchain technology can help improve the security of telesurgery by using NFTs to validate the identities of surgeons involved, adding a crucial layer of protection. NFTs could also be utilized to track specific surgical equipment, much like their current use tracking blood bank specimens, with each surgical tool assigned to a unique token. The usage history of a surgical device could be incorporated into its token and the blockchain would allow to trace the device's activity. Early adoption of NFT and blockchain systems in robotic surgical systems could prove vital to their ongoing development and integration into the global surgical tool kit.

Although there seems to be a plethora of uses for NFTs and blockchain in surgery, from tracking the use of surgical devices to organizing patient data, idealism must be balanced with practicality. In other words, current issues, and the ability of these transformative technologies to solve these issues, must be clearly defined before any healthcare system is dismantled and rebuilt on the blockchain, with additional consideration into costs of initiation and maintenance. NFTs and blockchain are creative technologies that hold promise to enhance the field of surgery. Taken together, blockchain and NFTs seem optimal for integration into fields requiring a decentralized and trustable chain of evidence, especially one where the integrity and security of historical data is crucial for success, such as the field of surgery.
